# Supplementary material for: Predicting interaction-specific protein–protein interaction perturbations by missense variants with MutPred-PPI
Source: bioRxiv. 2025 Dec 23:2025.12.20.695738. Preprint. [Version 1] doi: 10.64898/2025.12.20.695738 (PMC12767517; doi:10.64898/2025.12.20.695738)
Supplement: Supplementary file 1 [file media-1.pdf]

Supplementary Information

Predicting interaction-specific protein–protein interaction perturbations by missense variants with MutPred-PPI

Ross Stewart[1], Florent Laval[2,3,4,5,6,7,8], Georges Coppin[2,3,4,6], Kerstin Spirohn-Fitzgerald[2,3,4], Maxime Tixhon[2,3,4,9], Tong Hao[2,3,4], Michael A. Calderwood[2,3,4], Matthew Mort[10], David N. Cooper[10], Marc Vidal[2,3], Predrag Radivojac[1,*]

[1]Khoury College of Computer Sciences, Northeastern University, Boston, MA, USA
[2]Center for Cancer Systems Biology (CCSB), Dana-Farber Cancer Institute, Boston, MA, USA
[3]Department of Genetics, Blavatnik Institute, Harvard Medical School, Boston, MA, USA
[4]Department of Cancer Biology, Dana-Farber Cancer Institute, Boston, MA, USA
[5]TERRA Teaching and Research Centre, University of Liège, Gembloux, Belgium
[6]Laboratory of Viral Interactomes, GIGA Institute, University of Liège, Liège, Belgium
[7]Laboratory of Molecular and Cellular Epigenetics, GIGA Institute, University of Liège, Liège, Belgium
[8]Donnelly Centre for Cellular and Biomolecular Research, University of Toronto, Toronto, ON, Canada
[9]Computational Biology and Bioinformatics, Université Libre de Bruxelles, Brussels, Belgium
[10]Institute of Medical Genetics, School of Medicine, Cardiff University, Cardiff, UK

[*]Corresponding author: Predrag Radivojac (predrag@northeastern.edu)

Contents

1 Detailed model architecture **1**
1.1 Input representation 1
1.2 Graph attention layers 1
1.3 Mutation processing 2
1.4 Prediction head 2

2 Variant repositories data sources **3**

3 Supplementary figures **5**

1 Detailed model architecture

1.1 Input representation

The input to the model consists of: a node feature matrix $\mathbf{X} \in \mathbb{R}^{n \times d}$, where n is the length of the concatenated sequences of proteins i and p, and $d = 1024$ represents the embedding dimension of ProtT5. The adjacency matrix $\mathbf{A} \in \{0,1\}^{n \times n}$ encodes residue contacts. For each variant, let $k \in \{1, \ldots, |i|\}$ be the residue index of the mutation in the interactor protein. We compute the mutation encoding $\tilde{\Delta\mathbf{x}}_k \in \mathbb{R}^d$ as the normalized difference between the mutant and wild-type embeddings at position k.

1.2 Graph attention layers

The node features are processed through two GAT layers. Each GAT layer computes updated node representations using multi-head attention:

$$\mathbf{h}_i^{(l+1)} = \sigma\left(\frac{1}{K}\sum_{k=1}^{K}\sum_{j\in\mathcal{N}_i}\alpha_{ij}^{k,(l)}\mathbf{W}^{k,(l)}\mathbf{h}_j^{(l)}\right) \quad (1)$$

where $\mathbf{h}_i^{(l)}$ is the representation of node i at layer l, $\mathbf{W}^{k,(l)}$ is the learnable weight matrix for attention head k at layer l, K is the number of attention heads, $\mathcal{N}_i$ represents the one-hop neighborhood of node i defined by the adjacency matrix, and $\alpha_{ij}^{k,(l)}$ are the attention coefficients for head k in layer l. The attention coefficients are computed as follows:

$$\alpha_{ij}^{k,(l)} = \frac{\exp\left(\text{LeakyReLU}\left(\mathbf{a}^{k,(l)T}[\mathbf{W}^{k,(l)}\mathbf{h}_i^{(l)} \parallel \mathbf{W}^{k,(l)}\mathbf{h}_j^{(l)}]\right)\right)}{\sum_{m\in\mathcal{N}_i}\exp\left(\text{LeakyReLU}\left(\mathbf{a}^{k,(l)T}[\mathbf{W}^{k,(l)}\mathbf{h}_i^{(l)} \parallel \mathbf{W}^{k,(l)}\mathbf{h}_m^{(l)}]\right)\right)} \tag{2}$$

where $\parallel$ denotes concatenation and $\mathbf{a}^{k,(l)}$ is a learnable attention vector. Specifically, our architecture uses the following:

$$\mathbf{H}^{(1)} = \text{ReLU}\left(\text{GAT}_1(\mathbf{X}, \mathbf{A}; \theta_1)\right) \in \mathbb{R}^{n\times 4h} \tag{3}$$

$$\mathbf{H}^{(2)} = \text{ReLU}\left(\text{GAT}_2(\mathbf{H}^{(1)}, \mathbf{A}; \theta_2)\right) \in \mathbb{R}^{n\times h/2} \tag{4}$$

where the first layer uses $K = 4$ attention heads with hidden dimension $h = 256$ (concatenated), and the second layer uses a single head.

1.3 Mutation processing

The normalized mutation encoding is processed through a dedicated neural network:

$$\mathbf{m} = f_{\text{mut}}(\tilde{\Delta\mathbf{x}}_k; \phi) = \text{MLP}_\phi(\tilde{\Delta\mathbf{x}}_k) \in \mathbb{R}^{h/2} \tag{5}$$

where f_{mut} is a multilayer perceptron (MLP) with one hidden layer, ReLU activation, and dropout:

$$\mathbf{z}_1 = \text{Dropout}_{0.1}(\text{ReLU}(\mathbf{W}_1\tilde{\Delta\mathbf{x}}_k + \mathbf{b}_1)) \tag{6}$$

$$\mathbf{m} = \mathbf{W}_2\mathbf{z}_1 + \mathbf{b}_2 \tag{7}$$

1.4 Prediction head

The final prediction combines the local structural context at the mutation site with the processed mutation encoding:

$$\mathbf{c} = [\mathbf{h}_k^{(2)} \parallel \mathbf{m}] \in \mathbb{R}^h \tag{8}$$

where $\mathbf{h}_k^{(2)}$ is the learned representation of the mutation site residue after two GAT layers. The probability of interaction loss is computed as:

$$\Pr(\text{interaction loss} \mid i, v, p) = \sigma\left(f_{\text{pred}}(\mathbf{c}; \psi)\right) \tag{9}$$

where f_{pred} is an MLP with two hidden layers:

$$\mathbf{o}_1 = \text{Dropout}_{0.1}(\text{ReLU}(\mathbf{W}_3\mathbf{c} + \mathbf{b}_3)) \in \mathbb{R}^{h/2} \tag{10}$$

$$\mathbf{o}_2 = \text{Dropout}_{0.1}(\text{ReLU}(\mathbf{W}_4\mathbf{o}_1 + \mathbf{b}_4)) \in \mathbb{R}^{h/8} \tag{11}$$

$$\text{logit} = \mathbf{W}_5\mathbf{o}_2 + \mathbf{b}_5 \in \mathbb{R} \tag{12}$$

The complete model can be summarized as follows:

$$\Pr(\text{interaction loss} \mid i, v, p) = \sigma\left(f_{\text{pred}}\left([\mathbf{h}_k^{(2)} \parallel f_{\text{mut}}(\tilde{\Delta\mathbf{x}}_k)]\right)\right) \tag{13}$$

where all parameters $\{\theta_1, \theta_2, \phi, \psi\}$ are learned through binary cross-entropy loss minimization.

2 Variant repositories data sources

To construct an interactome network, we downloaded all interacting human protein pairs with physical binding evidence from BioGRID Release 4.4.244. We then gathered variants from multiple repositories for analysis. From ClinVar [1] (January 2, 2025 release), we collected pathogenic (P/LP) and benign (B/LB) missense variants with at least one review star, as well as all missense variants of uncertain significance (VUS). All cancer-associated missense variants, recurrence counts, and gene classifications (oncogene vs. tumor suppressor gene, TSG) were downloaded from COSMIC [2] v101 (accessed under academic research terms). To define the oncogene and TSG sets, we retained only genes assigned to a single class. Disease-linked missense variants from HGMD [3] Professional 2025 were downloaded and mapped to amino acid substitutions using ANNOVAR [4], retaining only "DM" variants while excluding "DM?" variants. Population variants were obtained from gnomAD [5] v4.1.0 across all chromosomes and mapped to amino acid substitutions using ANNOVAR. Allele frequencies (AFs) were assigned using the GroupMax flag. Finally, for the two *de novo* variant datasets linked to neurodevelopmental disorders: one (NDD dataset [6]) contained case and control variants from autism spectrum disorder (ASD), intellectual disability, schizophrenia, and epileptic encephalopathy, and the other (ASD-specific dataset [7]) contained case variants specifically associated with ASD. Detailed dataset statistics are available in Supplementary Table S1. For each variant group, we report the number of unique proteins, protein pairs, variants, triplets (variant-partner combinations), and mean partners (the mean number of partners tested per variant).

Table S1: Dataset statistics for variant repositories

Dataset	Proteins	Pairs	Variants	Triplets	Mean Partners
ClinVar					
Rare Benign	630	559	616	2,054	3.3
Benign	1,964	2,302	4,217	20,057	4.8
Pathogenic	1,409	1,572	9,254	72,143	7.8
VUS	2,609	3,604	67,711	313,193	4.6
COSMIC					
Single-occurrence	2,447	3,136	120,559	431,607	3.6
Recurrence ≥ 2	2,425	3,717	37,336	155,962	4.2
Recurrence ≥ 4	2,091	2,837	6,725	35,895	5.3
Recurrence ≥ 8	1,310	1,436	2,090	13,678	6.5
Recurrence ≥ 16	700	695	834	6,304	7.6
Recurrence ≥ 32	354	355	325	2,704	8.3
COSMIC (Oncogenes)					
Single-occurrence	110	220	2,682	14,079	5.2
Recurrence ≥ 2	671	1,431	2,290	12,873	5.6
Recurrence ≥ 4	630	1,217	945	5,416	5.7
Recurrence ≥ 8	576	980	493	2,923	5.9
Recurrence ≥ 16	374	599	286	1,695	5.9
Recurrence ≥ 32	238	343	156	927	5.9
COSMIC (Tumor Suppressor Genes)					
Single-occurrence	143	286	3,501	21,441	6.1
Recurrence ≥ 2	943	1,738	2,270	20,835	9.2
Recurrence ≥ 4	902	1,442	659	7,632	11.6
Recurrence ≥ 8	630	906	227	2,947	13.0
Recurrence ≥ 16	269	381	81	1,017	12.6
Recurrence ≥ 32	98	132	24	282	11.8
HGMD					
All	1,391	1,545	3,620	21,458	5.9
gnomAD					
All	1,073	1,118	249,316	678,231	2.7
AF $\leq$ 1e-6	431	350	10,503	22,765	2.2
1e-6 $<$ AF $\leq$ 1e-5	456	371	7,653	16,064	2.1
1e-5 $<$ AF $\leq$ 1e-4	464	380	16,867	34,898	2.1
1e-4 $<$ AF $\leq$ 1e-3	404	316	3,259	6,548	2.0
1e-3 $<$ AF $\leq$ 1e-2	294	215	568	1,072	1.9
1e-2 $<$ AF	183	131	220	377	1.7
Neurodevelopmental Disorders					
Case	3,359	5,694	1,065	8,338	7.8
Control	2,157	2,744	307	3,045	9.9
Autism Spectrum Disorder					
Case	558	591	332	5,831	17.6

3 Supplementary figures

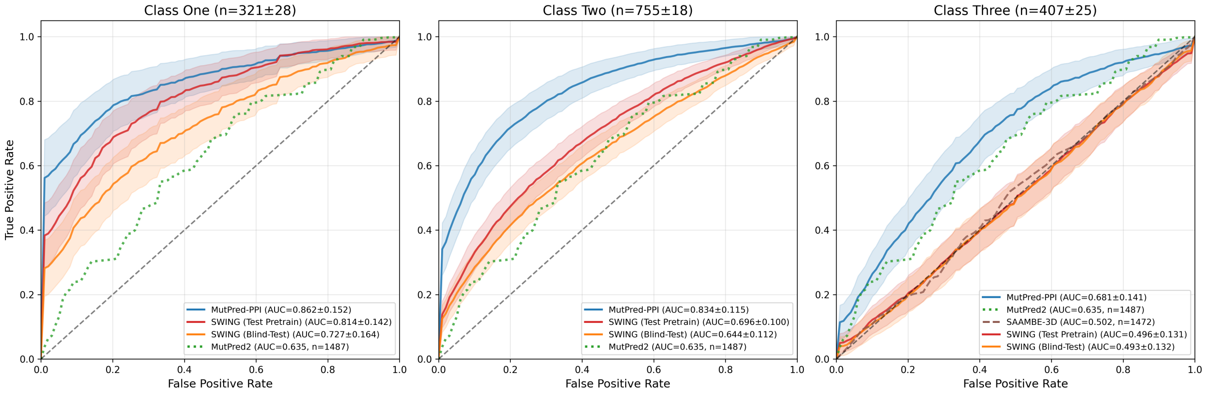

Figure S1: Cross-validation performance on Mendelian variant training data (n = 1,487). Solid lines show mean ROC curves across 30 iterations of 10-fold group cross-validation; shaded regions indicate ± 1 standard error of the mean computed across the 10 folds. Classes one, two, and three represent predictions with both proteins shared, one protein shared, and neither protein shared between training and test data, respectively. MutPred2 predictions are shown constant across test classes (partner-agnostic). Subplot titles show test set sizes. Proxy methods (dashed and dotted lines) display sample sizes in parentheses. Curves with $n < 30$ were excluded.

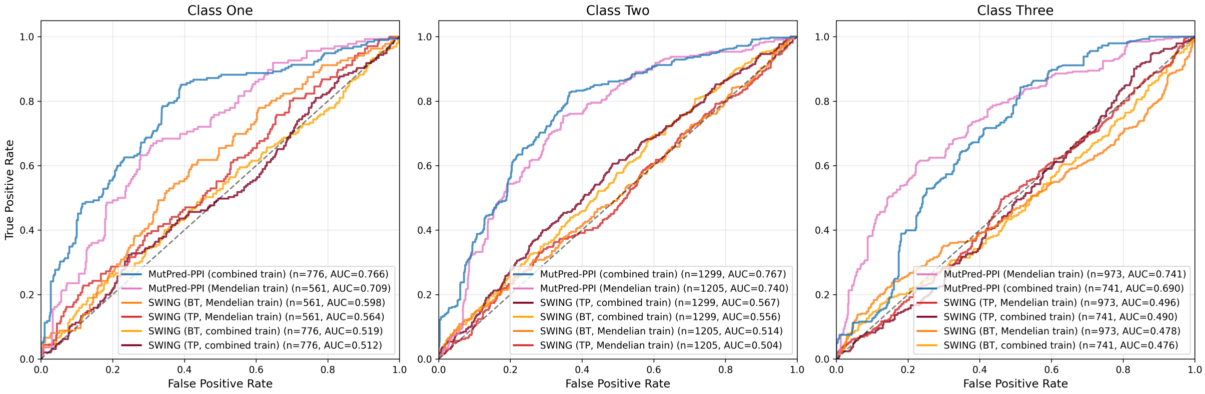

(a) VarChAMP dataset

Figure S2: Blind-test ROC curves on the VarChAMP dataset (n = 2,739–2,816) comparing training on both Mendelian and population variants vs. Mendelian variants only. Classes one, two, and three represent predictions with both proteins shared, one protein shared, and neither protein shared between training and test data, respectively. TP: test pretrain, BT: blind-test.

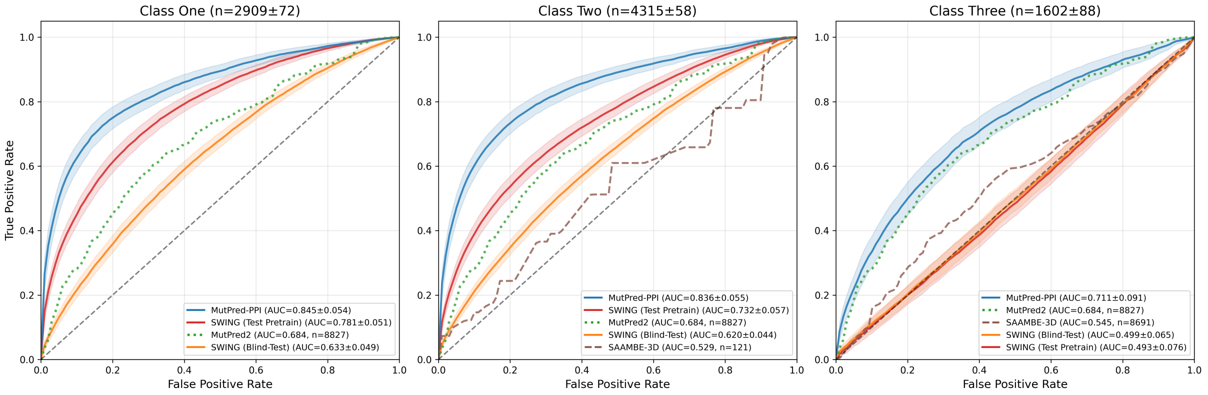

Figure S3: ROC curves of cross-validation predictions on the combined Mendelian, population, and VarChAMP datasets ($n = 8{,}827$). Solid lines show mean ROC curves across 30 iterations of 10-fold group cross-validation; shaded regions indicate ± 1 standard error of the mean computed across the 10 folds. Classes one, two, and three represent predictions with both proteins shared, one protein shared, and neither protein shared between training and test data, respectively. MutPred2 predictions are shown constant across test classes (partner-agnostic). Subplot titles show test set sizes. Proxy methods (dashed and dotted lines) display sample sizes in parentheses. Curves with $n < 30$ were excluded.

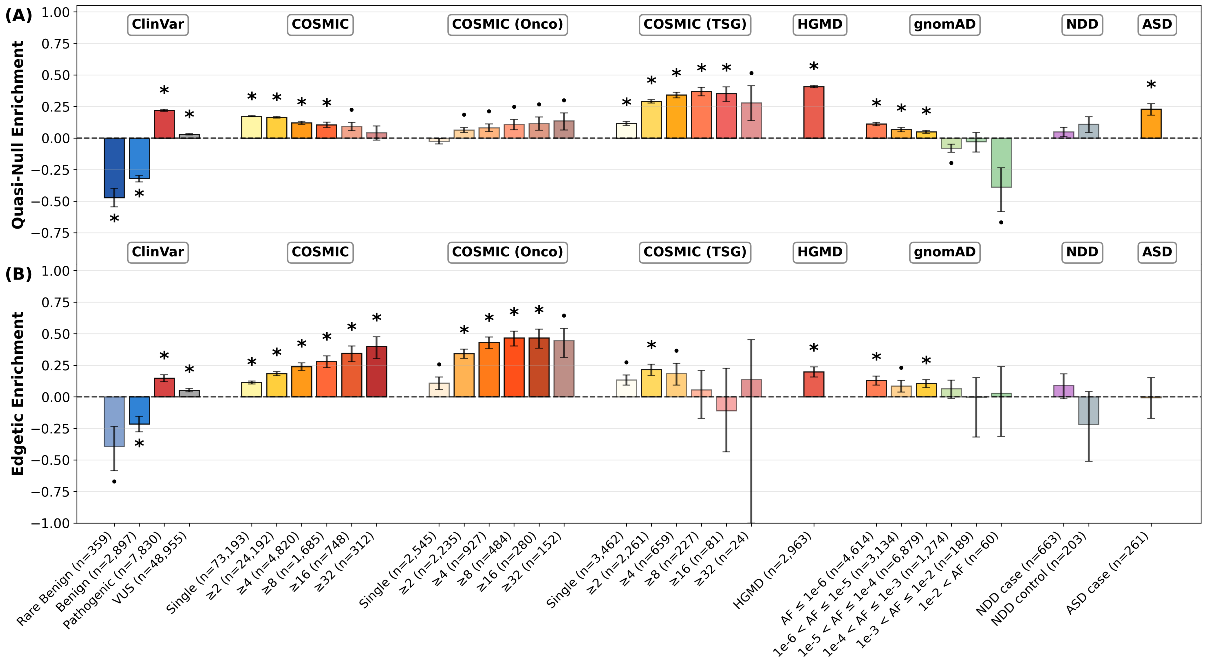

Figure S4: Partner-controlled edgotype enrichment with three partners sampled per variant. For each of 100,000 bootstrap iterations, three partner proteins were randomly sampled for each variant; variants with fewer than three partners were excluded. COSMIC, COSMIC (Onco), and COSMIC (TSG) are binned by recurrence; gnomAD is binned by allele frequency (AF). Rare benign variants are defined as AF $\leq$ 1%. Error bars indicate 68% confidence intervals. Asterisks indicate statistical significance with Bonferroni correction ($\alpha = 0.05$, $n_{\text{tests}} = 64$). Dots indicate significance at the uncorrected $\alpha = 0.05$ level. (A) Quasi-null enrichment. (B) Edgetic enrichment. VUS: variants of uncertain significance.

References

[1] M. J. Landrum, J. M. Lee, M. Benson, *et al.*, "ClinVar: Improving access to variant interpretations and supporting evidence," *Nucleic Acids Research*, vol. 46, no. D1, pp. D1062–D1067, 2018. DOI: 10.1093/nar/gkx1153.

[2] J. G. Tate, S. Bamford, H. C. Jubb, *et al.*, "COSMIC: The catalogue of somatic mutations in cancer," *Nucleic Acids Research*, vol. 47, no. D1, pp. D941–D947, 2019. DOI: 10.1093/nar/gky1015.

[3] P. D. Stenson, M. Mort, E. V. Ball, *et al.*, "The Human Gene Mutation Database (HGMD®): Optimizing its use in a clinical diagnostic or research setting," *Human Genetics*, vol. 139, no. 10, pp. 1197–1207, 2020. DOI: 10.1007/s00439-020-02199-3.

[4] K. Wang, M. Li, and H. Hakonarson, "ANNOVAR: Functional annotation of genetic variants from high-throughput sequencing data," *Nucleic acids research*, vol. 38, no. 16, e164–e164, 2010. DOI: 10.1093/nar/gkq603.

[5] S. Chen, L. C. Francioli, J. K. Goodrich, *et al.*, "A genomic mutational constraint map using variation in 76,156 human genomes," *Nature*, vol. 625, no. 7993, pp. 92–100, 2024. DOI: 10.1038/s41586-023-06045-0.

[6] V. Pejaver, J. Urresti, J. Lugo-Martinez, *et al.*, "Inferring the molecular and phenotypic impact of amino acid variants with MutPred2," *Nature Communications*, vol. 11, no. 1, p. 5918, 2020. DOI: 10.1038/s41467-020-19669-x.

[7] J. M. Fu, F. K. Satterstrom, M. Peng, *et al.*, "Rare coding variation provides insight into the genetic architecture and phenotypic context of autism," *Nature Genetics*, vol. 54, no. 9, pp. 1320–1331, 2022. DOI: 10.1038/s41588-022-01104-0.
